# Response to comment on *Have we been qualifying measurable residual disease correctly?*

**DOI:** 10.1038/s41375-023-02088-4

**Published:** 2023-11-21

**Authors:** Junren Chen

**Affiliations:** 1 State Key Laboratory of Experimental Hematology, National Clinical Research Center for Blood Diseases, Haihe Laboratory of Cell Ecosystem, Institute of Hematology & Blood Diseases Hospital, Chinese Academy of Medical Sciences & Peking Union Medical College, Tianjin, China; 2 Tianjin Institutes of Health Science, Tianjin, China

**Keywords:** Acute lymphocytic leukaemia, Paediatrics

## To the Editor:

Professor Morley was correct in pointing out in our Perspective we focused on analysis of Poisson noise, a type of sampling error [1]. Indeed, it is sometimes useful to distinguish between errors in sampling cells from a sub-population and errors in sampling a sub-population from the entire population of cells. The underlying mechanisms for these two types of sampling errors could be similar. Although in our Perspective we did not dwell on spatial distribution of leukaemia cells, we now caution an uneven spatial distribution could also be due to Poisson noise, as exemplified by R. D. Clarke’s classic spatial analysis of the distribution of flying-bomb attacks in London during WWII [2]. It is also important to recognise any sampling error at the cell-count level cannot be salvaged by lysis of the sampled cells for subsequent nucleic acid analysis such as quantitative real-time polymerase chain reaction (RT-qPCR) or next-generation sequencing (NGS).

Some leukaemia treatment protocols do call for measurable residual disease (MRD)-testing at a time when collecting a large number of bone marrow cells is not always feasible [3, 4]. The ideal scenario is of course having a multi-parameter flow cytometry (MPFC)-based MRD-test declared positive only if ≥5 × 10E+5 cells are analysed and if ≥50 cells are positive, but oftentimes physicians need to make decisions under non-ideal conditions. Should consideration of sampling errors affect the treatment plan for a person? We believe it should, but not until more validation studies are conducted. We agree a global platform such as EuroMRD would be the right venue for advancing proper usage of MRD-tests.

Professor Morley argued that decisions based on the conventional MRD values will optimise treatment for the group as a whole. We disagree. The conventional MRD value is not the mean or median estimate of true MRD. Rather, when conventional MRD is zero, it is the optimist’s rosy estimate of true MRD assuming all such patients have near-zero leukaemia cell. Making decisions based on such false optimism would not optimise treatment for the group as a whole, not to mention some of the individual patients. Perhaps even a hospital administrator should consider including ${{{{{{\rm{MRD}}}}}}}_{{{{{{\rm{worst}}}}}}\_{{{{{\rm{case}}}}}}}$ as one of her benchmarks for evaluating treatment efficacy *as a whole*.

**Fig. 1:**
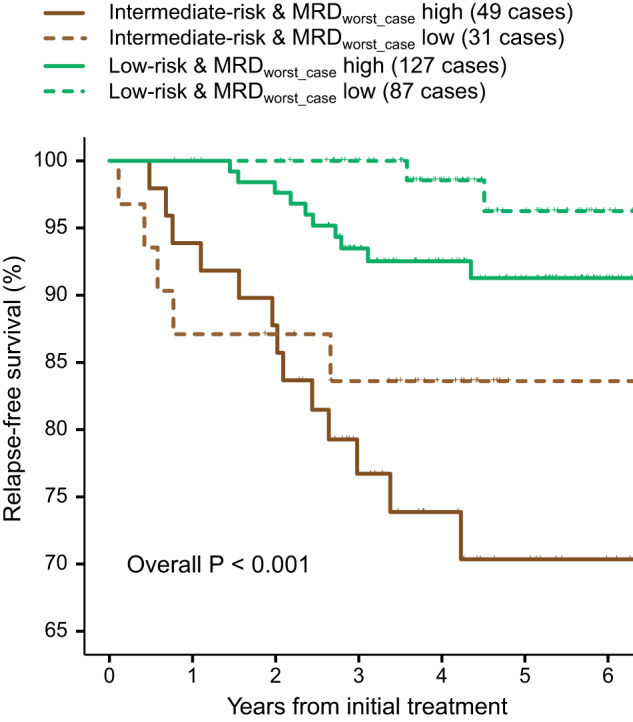
Risk-stratification based on joint consideration of estimated relapse risk at diagnosis and ${{{{{{\rm{MRD}}}}}}}_{{{{{{\rm{worst}}}}}}\_{{{{{\rm{case}}}}}}}$ on day 19 when ${{{{{{\rm{MRD}}}}}}}_{{{{{{\rm{conventional}}}}}}}$ on day 19 was $< {{0.01}} {{\%}}$. The study cohort is as described previously [1].

Finally, in our cohort of children with acute lymphoblastic leukaemia treated on CCCG-ALL-2015, ${{{{{{\rm{MRD}}}}}}}_{{{{{{\rm{worst}}}}}}\_{{{{{\rm{case}}}}}}}$ identified sub-groups of children with poorer relapse-free survival even though their conventional MRD-test results were nearly all zero (Fig. 1). Nonetheless, like others we avoid relying on composite end points that could have heterogeneous make-up of adverse events [5]. In our opinion, it is crucial for an MRD researcher to clarify the impact of an MRD-testing result on the cumulative incidence of relapse itself.
